# A novel *PHKA2* mutation in a Chinese child with glycogen storage disease type IXa: a case report and literature review

**DOI:** 10.1186/s12881-019-0789-8

**Published:** 2019-03-29

**Authors:** Junling Fu, Tong Wang, Xinhua Xiao

**Affiliations:** Department of Endocrinology, Chinese Academy of Medical Sciences and Peking Union Medical College, Peking Union Medical College Hospital, Beijing, China

**Keywords:** Glycogen storage disease type IX, Phosphorylase b kinase 2, *PHKA2* gene, Hypoglycaemia

## Abstract

**Background:**

*PHKA2* gene mutations can cause liver phosphorylase kinase (PhK) deficiency, resulting in glycogen storage disease type IXa (GSD IXa). Elevated liver transaminase levels and liver enlargement are the most frequent phenotypes of GSD IXa. However, whether the phenotypes are applicable to Chinese patients remains unclear.

**Case report:**

A boy aged 2 years and 8 months with a history of episodic fatigue and weakness since he was 2 years old was referred to our endocrinology clinic. Apart from symptomatic ketotic hypoglycemic episodes (palpitation, hand shaking, sweating, etc.), no abnormalities of liver transaminase levels or liver size were found. To identify the aetiology of his clinically diagnosed hypoglycaemia, the proband and his parents were screened for *PHKA2* gene mutations by next-generation sequencing. A heterozygous mutation (c.2972C > G, p.G991A) in *PHKA2* was found in the proband and his mother. Twenty-one Chinese cases with GSD IXa have been reported in the literature to date, and elevated liver transaminase levels (95%) and liver enlargement (91%) are the most frequent phenotypes of GSD IXa in Chinese patients. Hypoglycaemia may be one of the early onset symptoms in infants with GSD IXa.

**Conclusions:**

This study enriches our knowledge of the *PHKA2* gene mutation spectrum and provides further information about the phenotypic characteristics of Chinese GSD IXa patients.

## Background

Glycogen storage diseases (GSDs) are inborn errors of glycogen metabolism characterized by an interruption of glycogen breakdown and/or accumulation of a harmful glycogen [1]. GSD type IX (GSD IX) is caused by a defect in phosphorylase b kinase (PhK), which activates glycogen phosphorylase and thus plays a key role in regulating the mobilization of glycogen to glucose [1]. PhK is composed of four different subunits: α, β, γ and δ. The subunits possess tissue-specific isoforms encoded by different genes [2]. The liver-specific isoforms of the α-, β- and γ-subunits are encoded by *PHKA2, PHKB* and *PHKG2*, respectively [2]. GSD IXa (MIM: 306000), due to a defect in the liver-particular α isoform (*PHKA2*), shows X-linked recessive inheritance characteristics [3]. The prevalence of GSD type IX is 1 in 100,000 [4], and GSD type IX accounts for approximately 25% of all types of GSD [4], while GSD IXa accounts for approximately 75% of total GSD IX cases. GSD IXa is characterized by hepatomegaly, chronic liver disease, hypoglycaemia, hyperketosis, hyperlipidemia, growth retardation and delayed motor development [5]. The diagnosis of GSD IX is complicated due to its rarity and the overlap of clinical phenotypes with other congenital diseases. For the technical constraints of the PhK measurement, molecular diagnosis is the key method for providing a definitive diagnosis of GSD IXa and is an effective way to avoid invasive procedures. Almost 107 mutations have been reported in the *PHKA2* gene to date. Here we report a novel mutation in the *PHKA2* gene and review the literature concerning the phenotypic and genotypic spectra of Chinese GSD IXa.

## Case presentation

A boy aged 2 years and 8 months with a history of episodic fatigue and weakness was admitted for the evaluation of symptomatic hypoglycemic episodes. The patient was the first child of unrelated Chinese parents. The boy was born by a full-term normal vaginal delivery after 40 weeks of gestation, with a birth weight of 3.3 kg and a birth length of 49 cm. His pre- and postnatal periods and developmental milestones were normal, and his parents and younger sister were healthy. The symptomatic hypoglycemic episodes were first noted when he was 2 years old. Physical examination showed a non-dysmorphic boy with a height of 97 cm (0SD~ + 1SD), a weight of 15.5 kg (0SD~ + 1SD), and no palpable liver enlargement. He displayed normal muscle strength according to the MRC scale.

The patient’s biochemical characteristics are listed in Table 1. Urine ketone bodies were positive. His liver size was normal on ultrasonography with no liver hyperechogenicity or hypoechogenicity. His electroencephalography, social life ability and intelligence development were normal. His Peabody developmental motor scale presented a motor delay [Gross Motor Quotient (78); Fine Motor Quotient (87); Total Motor Quotient (83)] [6].

**Table 1:** Biochemical characteristics

Biochemical parameters	Value	Normal range
Glucose (mmol/L)	2.8	3.89–6.11
β-hydroxybutyric acid (mmol/L)	2.71	0.02–0.27
ALT (U/L)	13	0–40
AST (U/L)	36	0–40
GGT (U/L)	10	11–50
TBIL (umol/L)	8.9	5.0–28.0
Albumin (g/L)	47.5	35–55
Creatinine (umol/L)	17	53–123
Cholesterol (mmol/L)	4.1	3.0–5.17
TG (mmol/L)	0.57	0.56–1.71
CK (U/L)	151	38–174
Lactate (mmol/L)	1.0	0.5–2

Abbreviation: *AST* aspartate aminotransferase, *ALT* alanine transaminase, *GGT* gamma-glutamyl transpeptidase, *TG* triglyceride. *TBIL* total bilirubin, *CK* creatine kinase

Consent and Ethics:

Written informed consent was provided by the parents. This study was approved by the ethics committee of Peking Union Medical College Hospital, China.

## Methods

### Genetic study

#### Next-generation sequencing

Peripheral blood samples (4 ml) of the proband and his family members were collected. FlexiGene DNA Kit (Qiagen, Hilden, Germany) was used to extract genomic DNA according to the instructions. A screen for genetic mutations and copy number variants of 4000 inherited diseases was performed by the SureSelect Target Enrichment System (Agilent, Santa Clara, CA, USA). NEXTSEQ 500 sequencer (Illumina, San Diego, CA, USA) was applied for high-throughput sequencing. We searched pathogenic genes by analyzing the bioinformatics of the gene sequences. Genome Analysis Tool Kit (GATK) and ANNOVAR were used to determine and annotate the small deletions/insertions and single nucleotide polymorphisms. After removing the duplicated reads, databases including HGMD (http://www.hgmd.cf.ac.uk/ac/index.php), ClinVar (https://www.ncbi.nlm.nih.gov/clinvar/), 1000 Genomes Project (http://www.1000genomes.org/) and ExAC (http://exac.broadinstitute.org) were used to remove polymorphisms.

#### Sanger sequencing for validation

**Fig. 1:**
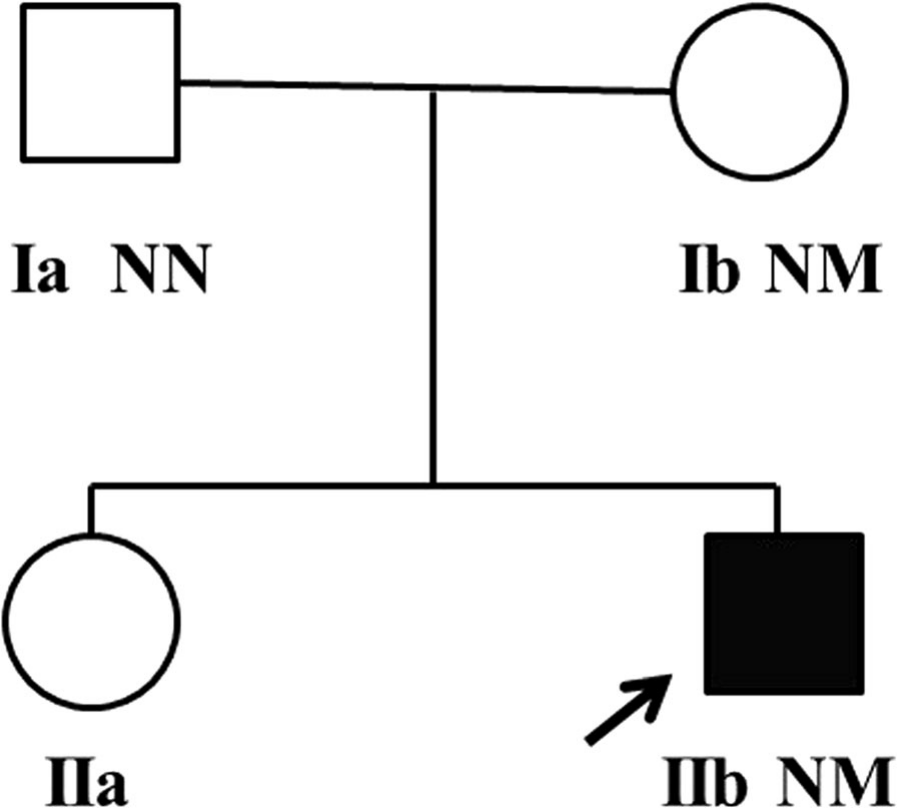
Pedigree of family in this study. Squares represent male family members, while circles represent female family members. Black symbol represents individual with GSD IXa, blank symbols represent normal individuals. Arrow indicates proband in the family (IIb). Variant carrier status present as N: Normal allele and M: Mutation. The sequence data displayed heterozygous mutation in *PHKA2* (c.2972C > G, p.G991A) in proband and his mother (Ib)

Sanger sequencing of *PHKA2* was conducted on DNA samples from the proband and his parents, as his little sister did not provide a blood sample for the analysis **(**Fig. 1**)**. Primers of *PHKA2* gene were designed by Primer Premier 5 software. Software PolyPhen2 (http://genetics.bwh.harvard.edu/pph2/), Mutation Taster (http://www.mutationtaster.org) and SIFT (http://sift.jcvi.org) were used to predict the pathogenicity of protein mutations.

#### Literature review

The literature search occurred in September 2018. We systematically identified all potentially relevant articles from three electronic databases: MEDLINE, PubMed and Web of Science. Search terms such as “glycogen storage disease type IX,” “glycogen storage disease,” “phosphorylase b kinase 2,” “*PHKA2* gene,” “case report,” and “Chinese” were used in various combinations and permutations across the databases.

## Results

### Molecular results

**Fig. 2:**
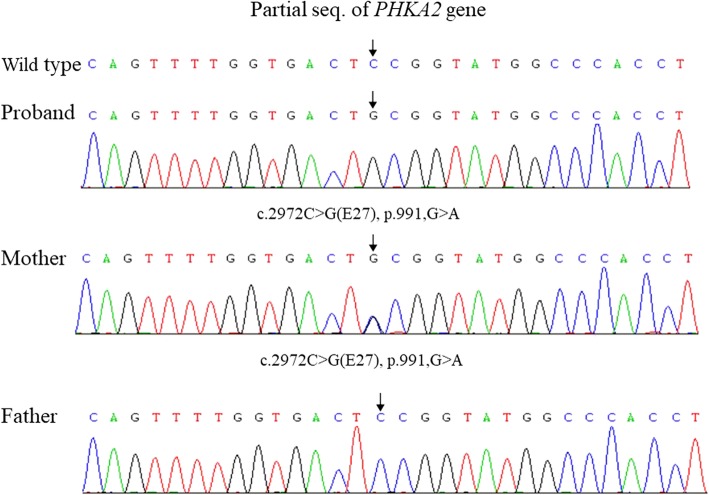
Results of genetic testing. A heterozygous mutation (p.G991A) at codon 991 of the *PHKA2* gene was revealed in the proband and his mother. No mutation was found in the proband’s father

Through data mining combined with genetic characteristics and clinical manifestations, we identified a novel heterozygous c.2972C > G mutation in *PHKA2* of the proband, which is considered to be the pathogenic mutation. The Sanger sequencing confirmation is shown in Fig. 2. The proband and his mother both carried a heterozygous c.2972C > G mutation. Thus, the mutation of the proband was derived from his mother. His mother was not affected because of the recessive X-linked transmission of the disorder. The novel mutation caused a protein change from G to A at p. G991A, which is located on exon 27 of *PHKA2*. The pathogenicity of the novel mutation was confirmed by 3 different programs: SIFT (0), PolyPhen-2 (0.989) and Mutation taster (disease-causing).

### Treatment and follow-up

After the accurate diagnosis, the patient was treated with uncooked cornstarch to avoid hypoglycaemia. After a 1 year follow-up, the boy showed normal growth and development and yielded no hypoglycaemia.

### Literature review

Twenty-one cases of Chinese GSD IXa have been reported to date [7–9]. The analysis of the PHKA2 gene was carried out in all the 21 cases and it identified a mutation in 19 cases. Szu-Ta Chen et al. [9] failed to identify a mutation in their two cases after sequencing exons 4, 6, 9, 26, 32 and 33, which cover 45.5% of the reported mutation sites and 48.8% of cases with GSD IXa. The genetic defects in the two cases may be located in uncommon sites. More specifically, 12 patients underwent liver biopsy, and 10 of them demonstrated consistent results with genetic testing. The phenotypic characteristics (Table 2) were dominated by elevated liver transaminase levels (95%) and liver enlargement (91%). Short stature (43%), hyperlipidemia (43%) and hypoglycaemia (38%) were less common. Delayed motor development was previously reported in only one case. The median age of onset and diagnosis of GSD IXa were 1.8 years old and 8.9 years old, respectively.

**Table 2:** Reports on Chinese patients with GSD IXa

Patients	Base change	Exon	Onset age	Diagnosis age	Elevated liver transaminase levels	Liver enlargement	Short stature	Hypoglycemia	Elevated of lactic acid	Hyperlipidemia	Delayed motor developmental	Liver biopsy
Reports 1 [7]												
1	c.133C > T	2	3	7	+	+	+	+	+	–	–	/
2	c.134G > A	2	2	3.8	+	+	–	+	+	–	–	/
3	c.237 + 1 G > T	2	2	9	+	+	+	–	+	+	–	+
4	c.338A > G	4	2	15	+	+	–	–	/	–	–	+
5	c.392G > A	4	6	17	+	+	–	–	/	–	–	+
6	c.407A > T	4	1	2.5	+	+	+	–	+	–	–	/
7	c.538G > A	6	5	7.1	+	–	+	–	–	+	–	/
8	c.884G > A	9	0.5	28	–	+	–	–	–	–	–	–
9	c.884G > A	9	10.5	11	+	–	–	+	–	+	–	+
10	c.884G > A	9	1.8	8.9	+	+	+	+	–	–	–	/
11	c.889G > A	9	1	23	+	+	–	–	/	+	–	+
12	c.1498C > T	15	1	2.5	+	+	–	–	–	–	–	/
13	c.1925C > G	18	0.8	2	+	+	–	+	–	–	–	/
14	c.2746C > T	25	0.7	7.8	+	+	–	+	/	+	–	/
15	c.2746C > T	25	3	9	+	+	–	+	–	–	–	+
16	c.2726_2727delTT	25	0.3	10	+	+	+	–	–	–	–	+
17	c.3377C > A	32	0.8	1.5	+	+	+	+	–	+	–	+
Reports 2 [8]												
18	–		3.5	4.5	+	+	+	–	/	+	–	+
19	–		/	1.25	+	+	+	–	/	+	–	+
Reports 3 [9]												
20	c.136delG	2	0.7	3	+	+	–	–	/	–	–	+
21	c.87029G > A	30	1.5	10	+	+	–	–	/	+	+	+

## Discussion and conclusions

In this study, we identified a novel mutation in *PHKA2* (c.2972C > G, p.G991A) in a patient with a relatively rare phenotype of GSD IXa, including hypoglycaemia and delayed motor development.

The intricacy of the enzyme structure results in heterogeneous clinical phenotypes. Overall, hepatomegaly, elevated liver transaminase levels, growth retardation, hypercholesterolemia, hypertriglyceridemia and hypoglycaemia can be present. Rare manifestations include elevated uric acid and lactic acid levels [5]. These clinical symptoms and biochemical manifestations ameliorate with age and even disappear in adulthood, with a spontaneous remission tendency [10]. The present patient manifested with symptomatic hypoglycemic episodes and motor delay at the age of 2 years old. However, common clinical manifestations, such as hepatomegaly and elevated liver transaminase levels, were not present. By contrast, Jiangwei Zhang et al. [8] reported a study including 17 GSD IXa patients, in which 16/17 patients displayed increased liver transaminase levels and 15/17 patients yielded liver enlargement. Importantly, by reviewing the previous literature, we found that patients who displayed severe hypoglycaemia were diagnosed as GSD IXa at an earlier age. Thus, hypoglycaemia may be one of the early symptoms of GSD IXa.

Through the literature review of 21 Chinese GSD IXa patients [7–9], elevated liver transaminase levels and liver enlargement were the relevant symptoms of GSD IXa. Delayed motor development was rare, to our knowledge; this is the second Chinese GSD IXa patient reported with delayed motor development. Importantly, the diagnosis of GSD IXa takes an average of 6 years, indicating that there are still challenges for the early diagnosis of GSD IXa, especially in regions where the enzymatic assay is impracticable. Certainly, genetic diagnosis provides benefits in the field.

GSD IXa is caused by mutations of the *PHKA2* gene located in Xp22.2–22.1. *PHKA2* contains 33 exons and encodes the liver-α subunit [11]. Until now, approximately 151 patients with 107 mutations of the *PHKA2* gene have been recorded by HGMD, including 7 splicing, 9 insertions, 29 deletion, 11 nonsense and 51 missense mutations. Currently, only sixteen mutations have been confirmed in Chinese patients [7–9]. In this case, we detected one novel missense mutation, which was not recorded in the NCBI dbSNP database, at an ultra-low frequency (0.0008) in the DYDF database. The mutation (p.G991A) resulted in a changeable protein structure and affected the nearbysplice site, which indicates that the mutation possibly causes skipping of the following exons. Therefore, we considered that this mutation may lead to the expression of an abnormal PhK α-subunit protein.

Previous studies have reported that patients with GSD IXa showed normal growth and development and normal liver transaminase levels despite their lack of treatment [12]. The patient in our study was treated with corn starch, which avoided recurrence of hypoglycaemia in the following year. Close monitoring is critical. Hence, the study may be useful for the future study of GSD IXa and provides further information about the phenotypic characteristics of Chinese GSD IXa patients.

## Abbreviation

GSD IXa: Glycogen storage disease type IXa
